# Memory impairment in older adults’ diversionary thoughts

**DOI:** 10.3389/fnagi.2015.00189

**Published:** 2015-10-21

**Authors:** Fátima Alves, Flávia Resende, Maria Salomé Pinho

**Affiliations:** Laboratory of Memory, Language and Executive Functions, Faculty of Psychology and Educational Sciences, University of CoimbraCoimbra, Portugal

**Keywords:** diversion paradigm, older adults, amnesic effect, autobiographical memories, free recall

## Abstract

The diversion paradigm was created in the context of explaining the effect of the instruction to forget some recently encoded material in the list-method of the directed forgetting paradigm. The current study of healthy older adults employed the diversion paradigm with two main goals: to determine whether thinking about an autobiographical memory interferes with the recall of recently encoded information and to explore whether the degree of forgetting depends on the temporal distance created by the diversionary thought. Ninety non-institutionalized Portuguese older adults (47 females and 43 males), aged 65–69 years, with education levels of between 3 and 6 years participated in this study. The exclusion criteria were as follows: presence of depressive symptomatology (assessed with the Geriatric Depression Scale-30) and global cognitive deterioration (assessed with the Mini–Mental State Examination). Concerning the diversion paradigm, one group was instructed to think about an autobiographical event (remembering one’s childhood home or the last party that one had attended) after studying one word list (List 1) and before viewing the second word list (List 2). After a brief distraction task, the participant had to recall the words from both of the studied lists. In the control group, the procedure was the same, but the diversionary thought was substituted by a speed reading task. The obtained results showed the amnesic effect of diversionary thought but did not show a greater degree of forgetting when the autobiographical events in the diversionary thoughts were temporally more distant. Considering the practical implications of these results, this study alerts us to the importance of promoting strategies that enable older adults to better remember important information and effectively forget irrelevant information.

## Introduction

Certainly, many of us have experienced boring or uninteresting contexts in our lives that have led our minds to journeys into the past, present or future that are full of idyllic images and that concern moments, problems or fantasies (Klinger, 1978). Who does not remember that time when the classroom teacher told us not to be distracted and to stop daydreaming? Actually, we were not distracted; we were only shifting our mind’s focus to a different mental context induced by the mind’s wandering or a mental diversion (Sahakyan and Kelley, 2002). Diversion thinking refers to off-task thoughts; in other words, our attention is dissociated from the current context and attached to the mind’s wandering context (Smallwood and Schooler, 2006). Returning to the classroom after our mini mental journey, we attempt to remember the information that we acquired before we began to wander, and we acknowledge the difficulty of recalling this information. The amnesic effect of daydreaming has occurred (Delaney et al., 2010).

Forgetting is commonly believed to be problematic, especially in the aging context. Its importance is underestimated, and it is viewed as a memory failure (Sheard and MacLeod, 2005). However, scientific evidence indicates that human beings have structures for suppressing irrelevant information, disallowing the buildup of information interference and improving learning and retrieval abilities (e.g., Hasher et al., 1999; Anderson and Craik, 2000). Forgetting is essential to an efficient memory system (Bjork et al., 2006), and it has been widely studied using the directed-forgetting paradigm (e.g., Bjork, 1970, 1989; Johnson, 1994; MacLeod, 1998). This paradigm has two procedures: the list method and the item method*.* In the item method, the forgetting instruction is given item-by-item (e.g., Basden and Basden, 1996; MacLeod, 1999; Sahakyan and Foster, 2009). By contrast, the list method employs the forgetting instruction after a whole list—generally, the first list—has been presented. The main result of these procedures shows contrasting memory performance for the to-be-forgotten items vs. the to-be-remembered items: lower recall is observed for the to-be-forgotten items in comparison with the recall level of the to-be-remembered items.

Sahakyan and Kelley (2002) developed the diversion paradigm to explain how participants in a directed forgetting paradigm with the list method comply with the instruction to forget some recently presented material. In this paradigm, after learning the first list, the participants are instructed to engage in diversionary thought (e.g., thinking about their parents’ home or imagining being invisible). With these instruction modifications that explicitly command the mind’s focus to another mental context, it was observed that both younger and older adults showed significant forgetting (Sahakyan et al., 2008). One explanation for the diversion paradigm effect (the impaired ability to retain the information acquired immediately before the diversionary thought) claims that diversionary thought begins a new mental context in which the items on the second list are encoded and that, in this way, the context of the first list study becomes quite different from the context of the memory test (Sahakyan and Delaney, 2005; Delaney et al., 2010). It is also important to observe that accepting the contextual account does not necessarily imply discarding the possibility of the inhibition intervention. The contextual mental shifts (diversionary thought) might be followed by the inhibition of the unwanted context in which events were encoded (Anderson, 2009).

According to Delaney et al. (2010), it is important not only to study how diversionary thought contributes to forgetting information—which the authors call the amnesic effect of diversionary thought—but also to estimate the magnitude of the diversionary thought effect depending on the mental distance (temporal, circumstantial, spatial) from the current moment. With this aim, the authors conducted two experiments with undergraduate students that included different diversionary thoughts about autobiographical memories: the students’ parents’ home vs. their current home (Experiment 1) and international vs. domestic vacations (Experiment 2). The study results showed worse recall of the first word list when the diversionary thought condition differed greatly from the participants’ real situations in both space and time (long-distance vacations; their parents’ home) in comparison with the condition in which participants thought about events that were current or nearer in space.

The present study employed the diversion paradigm developed by Delaney et al. (2010) in a sample of healthy older adults. The study has two aims: to determine whether thinking about an autobiographical memory interferes with the recall of recently encoded information and to explore whether the temporal distance implied by diversionary thought has an effect on the level of forgetting, specifically, with older thought events leading to more forgetting. We were specifically interested in learning whether thinking about autobiographical memories related to recent events (the last party the participant had attended) triggered less forgetting than did personal memories concerning a distant event (the participants’ childhood home).

## Materials and Methods

### Subjects

Ninety community-dwelling older adults have voluntarily participated in this study. They were recruited through snowball sampling. The participants were assigned to one of the following conditions: experimental (two diversionary thought tasks, *N* = 30 each) or control (speed reading task, *N* = 30). Participants living in their childhood home were excluded from this study, as were those with a performance of more than 1 SD below the normal score on the Mini–Mental State Examination (MMSE; Folstein et al., 1975; Portuguese norms by Morgado et al., 2009) and the Vocabulary subtest of the Wechsler Adult Intelligence Scale 3rd edition; Wechsler, 2008). To rule out the presence of depressive symptomatology, participants with a score above 10 points on the Geriatric Depression Scale (GDS 30 item version; Yesavage et al., 1983; Portuguese adaptation and norms by Barreto et al., 2008) were excluded from the study. This study was approved by the Scientific Council of the Faculty of Psychology and Educational Sciences of the University of Coimbra, and verbal informed consent was obtained from participants prior to the session.

### Materials

Delaney et al. (2010) diversion paradigm was adapted to Portuguese older adults. Two lists were created, each with 16 unrelated concrete Portuguese nouns selected from the Corpus for European Portuguese norms (Nascimento et al., 2009). Written frequency of the stimuli was medium to high according to the same norms. The words were read sequentially, keeping a card with the word visible, at a rate of 5 s per word. Each list served as List 1 and List 2 an equal number of times. The experimental condition concerning the diversion paradigm included two diversionary thought tasks: the participants’ childhood home (old event condition) and the last party they had attended (recent event condition). The design of this experiment was a between-subjects design.

### Procedure

The experiment began with the administration of the diversion paradigm (Delaney et al., 2010). All participants were tested individually and instructed to study two word lists (List 1 and List 2) for a later memory test. After studying List 1, the participants in each experimental group were asked to perform, within 45 s, one of the two diversionary thought tasks, whereas those in the control group performed a speed reading task (reading an excerpt about a collage technique aloud as quickly as possible while ignoring its content). The text selected for the reading control task did not include any of the words on the word lists. In the diversionary thought task, participants were asked to remember their childhood home, imagining themselves there and describing their home aloud (old event condition) or doing the same for the last party they had attended (recent event condition) in accordance with their condition assignments. Then, all participants studied the second list of words followed by an arithmetic filler task of backwards counting for 90 s. At the end, the participants were asked to freely recall the maximum number of words from each list on separate sheets of paper. First, they recalled the words from List 1 and then the words from List 2. The amount of time allotted for each list recall was 80 s. A post-experiment questionnaire concerning the diversionary thought (e.g., How often do you remember/think about your childhood home? Apart from the current situation, how much time had elapsed since the last time you had thought about or remembered your childhood home?) was applied. Later, the participants were also administered the MMSE to screen for their general cognitive functioning, the Vocabulary subtest of the Wechsler Adult Intelligence Scale 3rd edition to briefly assess their verbal skills, and the GDS 30 to evaluate the presence of depressive symptomatology. As the exclusion criteria were also based on the results on these tests, 9 subjects were eliminated from the present study. This procedure left a total of 90 participants.

### Statistical Analysis

Statistical analysis was performed using the Statistical Package for the Social Sciences (SPSS) Version 18 for Windows (IBM, New York, USA). The level of significance adopted for all the statistical comparisons reported was set at *p* < 0.05. We computed a one-way analysis of variance (ANOVA) for unrelated samples for the comparisons between the control and experimental conditions concerning the results on the MMSE, GDS 30 and Vocabulary test, and the same statistical test was calculated with condition (diversionary thought about an old event, diversionary thought about a recent event, and no diversionary thought) as the independent variable for the comparison concerning the words recalled from List 1 and List 2 in each experimental condition and control condition.

## Results

Ninety non-institutionalized healthy older adults (47 females and 43 males) aged 65–69 years (*M* = 66.90, *SD* = 1.53), with education levels between 3 and 6 years, participated in this study.

The scores on the tests that were administered to assess the exclusion criteria for participants in this study are displayed in Table 1. The participants in the experimental conditions and in the control condition did not differ: *F*_(2,87)_ = 2.74, *MSE* = 4.900, *p* = 0.070, ${\text{η}}_{p}^{2}$ = 3.195 for the MMSE; *F*_(2,87)_ = 1.60, *MSE* = 13.756, *p* = 0.208, ${\text{η}}_{p}^{2}$ = 0.059 for the GDS 30 items; and *F*_(2,87)_ = 0.57, *MSE* = 46.344, *p* = 0.568, ${\text{η}}_{p}^{2}$ = 0.013 for the Vocabulary test.

**Table 1:** **Cognitive status, depressive symptomatology and vocabulary scores of the participants in the experimental and control conditions**.

	Experimental conditions				Control condition	
	The childhood home		The last party			
	*M*	*SD*	*M*	*SD*	*M*	*SD*
MMSE	27.63	1.35	28.43	1.33	28.13	1.33
GDS-30	6.70	2.26	5.80	2.02	6.53	1.93
Vocabulary	37.27	9.35	38.77	8.39	39.73	9.28

The mean proportions of words recalled from List 1 and List 2 in each experimental condition (old event: the childhood home, recent event: the last party) and control (no diversionary thought) are presented in Table 2.

**Table 2:** **Proportion of correct recall of both lists for experimental and control conditions**.

	List 1		List 2	
	*M*	*SD*	*M*	*SD*
**Experimental conditions**
The childhood home	0.14	0.09	0.22	0.11
The last party	0.13	0.07	0.21	0.15
**Control condition**	0.20	0.09	0.21	0.15

The ANOVA results indicated that condition significantly influenced the level of recall for List 1, *F*_(2,87)_ = 6.44, *MSE* = 0.045, *p* = 0.003, ${\text{η}}_{p}^{2}$ = 0.124. Games-Howell* post hoc* tests revealed significant differences between the old event (childhood home) condition and the control condition (*p* = 0.020) and between the recent event (last party) condition and the control condition (*p* = 0.008) but not between the old event condition and the recent event condition (*p* = 0.978). Thus, the amnesic effect of the diversionary thought was achieved. However, we did not find an effect of the event temporal distance produced by the diversionary thought, as the recall between the two experimental conditions did not differ. Concerning the List 2 recall, the ANOVA result was not statistically significant, *F*_(2,87)_ = 1.65, *MSE* = 0.023, *p* = 0.198, ${\text{η}}_{p}^{2}$ = 0.037, suggesting that the participants’ recall levels were equal in the three conditions.

## Discussion

In this study, the participants in both diversionary thought conditions recalled fewer words from List 1 than did participants in the condition with a reading speed task (control condition). Thus, remembering a personal past event produced recall impairment with the first studied word list, i.e., the expected amnesic effect of diversionary thought was achieved. The temporal distance of the diversionary thought (old event *vs* recent event conditions) did not influence recall; that is, the difference in List 1 recall levels between the two diversionary thought conditions did not reach statistical significance.

The first result concerning the amnesic effect of diversionary thought is in accordance with a previous study of the diversion paradigm in a sample of undergraduate students (Delaney et al., 2010) and with a directed forgetting paradigm employing the list method (Sahakyan et al., 2008) in a sample of younger and older adults. The explanation for this amnesic effect was not addressed in the present study, although context change (i.e., due to the contextual change induced by the diversionary thought, the context of encoding List 1 differed from the context of the memory test) has emerged in the literature as the strongest explanation (Sahakyan and Kelley, 2002; Sahakyan and Delaney, 2005; Delaney et al., 2010). The temporal distance of the diversionary thought (old event: the childhood home *vs* recent event: the last party) does not appear to differentially influence the recall of previously encoded information. To the best of our knowledge, there is no published study on whether the temporal distance created by a diversionary thought influences older adults’ forgetting. The result obtained differs from research with undergraduate students that suggests that diversionary thoughts affect the degree of forgetting information (Delaney et al., 2010): when the distance between the present moment and the past remembered event is longer, more forgetting occurs. The analysis of the participants’ responses to the post-experiment questionnaire, specifically the responses to the question about the last time they had thought about their childhood home/the last party that they had attended, indicated that the participants in the old event condition had retrieved memories of their childhood home more recently than the participants in the recent event condition had remembered the last party that they had attended. In future studies, the temporal distance of the diversionary thought should be further controlled. The study of these issues will be useful from a practical perspective to promote strategies that enable older adults to better remember important information and more effectively forget irrelevant information, at least immediately after encoding this information. The assessment of executive functions constitutes another limitation of this study, as these functions are strongly related to memory functioning and its decline occurs frequently in older people (e.g., Schaie and Willis, 2009).

The amnesic effect of diversionary thought that was achieved in the present study suggests that this effect is also found in normal aging. Given that remembering a past personal event can have a negative impact on the recall of recently encoded information, training that improves older adults’ attentional strategies becomes relevant. This training could help older people remember relevant information and forget unimportant information.

## Conflict of Interest Statement

The authors declare that the research was conducted in the absence of any commercial or financial relationships that could be construed as a potential conflict of interest.
